# Supplementary material for: Contemporary income inequality outweighs historic redlining in shaping intra-urban heat disparities in Los Angeles
Source: Nat Commun. 2025 May 28;16:4950. doi: 10.1038/s41467-025-59912-x (PMC12119888; doi:10.1038/s41467-025-59912-x)
Supplement: Supplementary file 1 — Supplementary Information [file 41467_2025_59912_MOESM1_ESM.pdf]

Supplementary Figures for

Contemporary income inequality outweighs historic redlining in shaping intra-urban heat disparities in Los Angeles

Authors: Anamika Shreevastava[1,2], Glynn Hulley[1], Sai Prasanth[1], TC Chakraborty[3], Diego Ramos Aguilera[4], Kelly T. Sanders[4], and Yi Yin[5]

[1]Jet Propulsion Laboratory, California Institute of Technology, CA, USA
[2]Environmental Science and Engineering, California Institute of Technology, CA, USA
[3]Pacific Northwest National Laboratory, WA, USA
[4]University of Southern California, CA, USA
[5]Department of Environmental Studies, New York University, NY, USA

Corresponding Author: ashreeva@caltech.edu

Table of Contents

Fig 1: ECOSTRESS LST for each season and time of day 1
Fig 2a: Diurnal evolution of LST across the HOLC grades in Spring 2
Fig 2b: Diurnal evolution of LST across the HOLC grades in Fall 2
Fig 2c: Diurnal evolution of LST across the HOLC grades in Winter 3
Fig 3: Cumulative LST distribution for Summer afternoon 3
Fig 4: Kolmogorov Smirnov test results 4
Fig 5: Median household income histogram and classification of rich, medium, and poor 5
Fig 6: Scatter plot between the percentage of households below poverty line and median household income 5
Fig 7a: LST boxplots by income classes and HOLC grades in Spring 6
Fig 7b: LST boxplots by income classes and HOLC grades in Summer 6
Fig 7c: LST boxplots by income classes and HOLC grades in Fall 7
Fig 7d: LST boxplots by income classes and HOLC grades in Winter 8
Fig 8: Seasonal hysteresis in vegetation indices 9
Fig 9: Seasonal hysteresis for each time of day 10
Fig 10: Air temperature distributions across the HOLC grades 11
Fig 11a: Principal Component Analysis 12
Fig 11b: PCA loading of each variable for first 3 PCs. 12
Fig 12: Population percentage of each of the individual ethnicities 14
Fig 13: Vulnerability Score distribution for each race/ethnicity 15
Fig 14: Population percentage sorted by HOLC grade and income 15
Fig 15: Analysis of AC usage data from multiple sources 16

Fig 1: ECOSTRESS LST for each season and time of day

(a)

Season / Time_of_day	Winter	Spring	Summer	Fall
PastMidnight	18	24	21	21
Morning	14	6	12	12
Afternoon	22	26	44	33
LateEvening	21	19	23	29

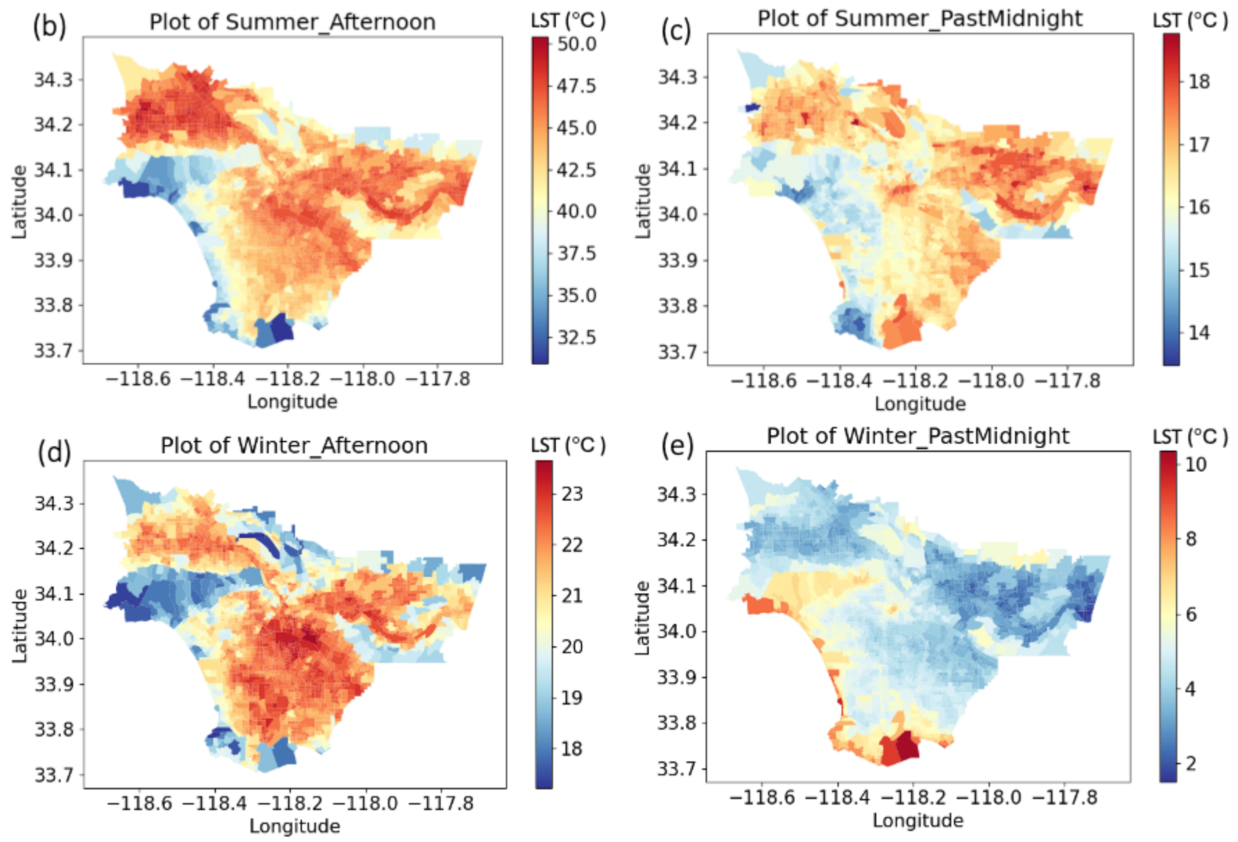

SI. Figure 1(a): Number of cloud-free ECOSTRESS scenes used for each season and time of day. For each of the 16 segments shown above, we create an average of the ECOSTRESS scenes while preserving the spatial structure. Subsequently, the raster image is resolved for each census tract to produce census specific LSTs. For example, (b) Time-averaged plot of ECOSTRESS LST for the time period between 12PM – 6 PM ("Afternoon") during the summer (c) Time-averaged plot of ECOSTRESS LST for the time-period between 12 AM– 6 AM ("past-midnight") during summer (d, e) Same as panels (b,c) but for winter. Census tract boundaries are based on 2020 shapefiles provided by the U.S. Census Bureau.

Fig 2a: Diurnal evolution of LST across the HOLC grades in Spring

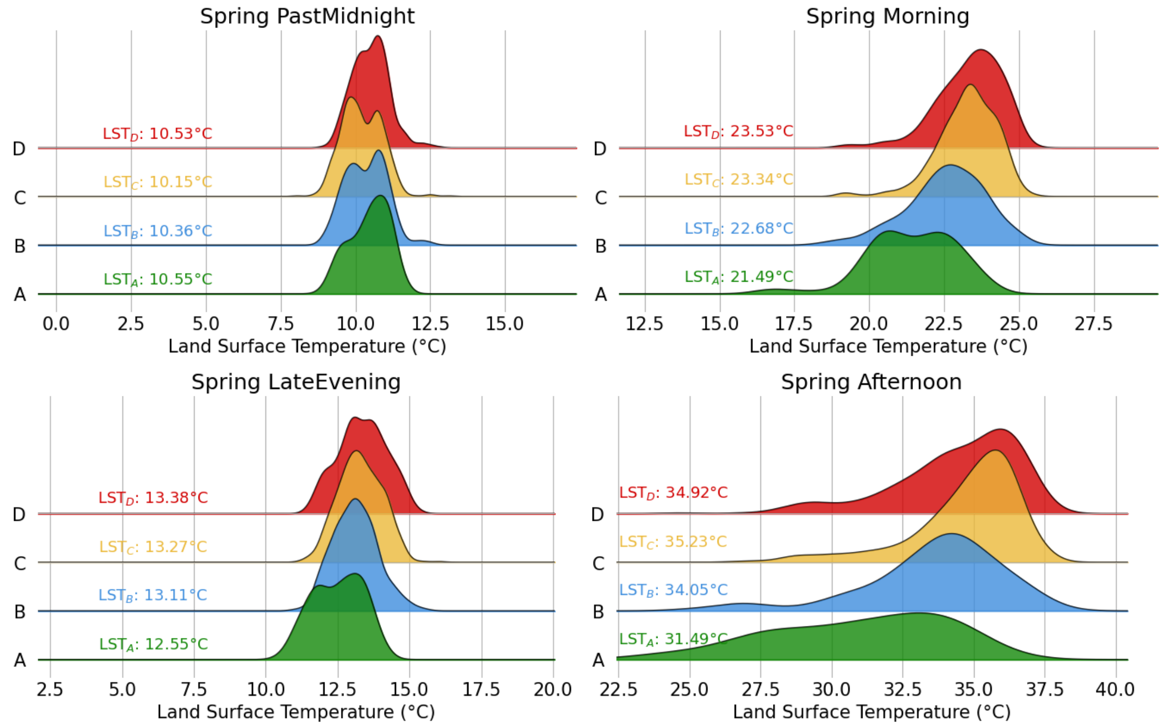

SI Figure 2b: Same as Figure 3 but for Spring seasons

Fig 2b: Diurnal evolution of LST across the HOLC grades in Fall

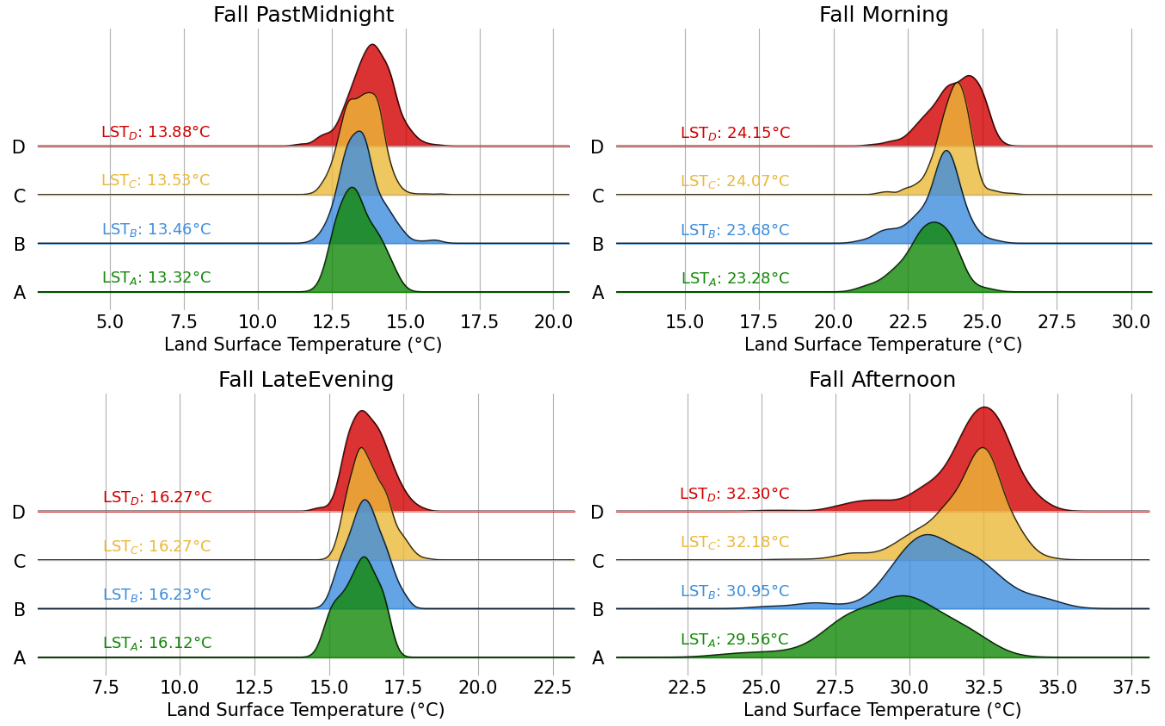

SI Figure 2b: Same as Figure 3 but for Fall seasons

Fig 2c: Diurnal evolution of LST across the HOLC grades in Winter

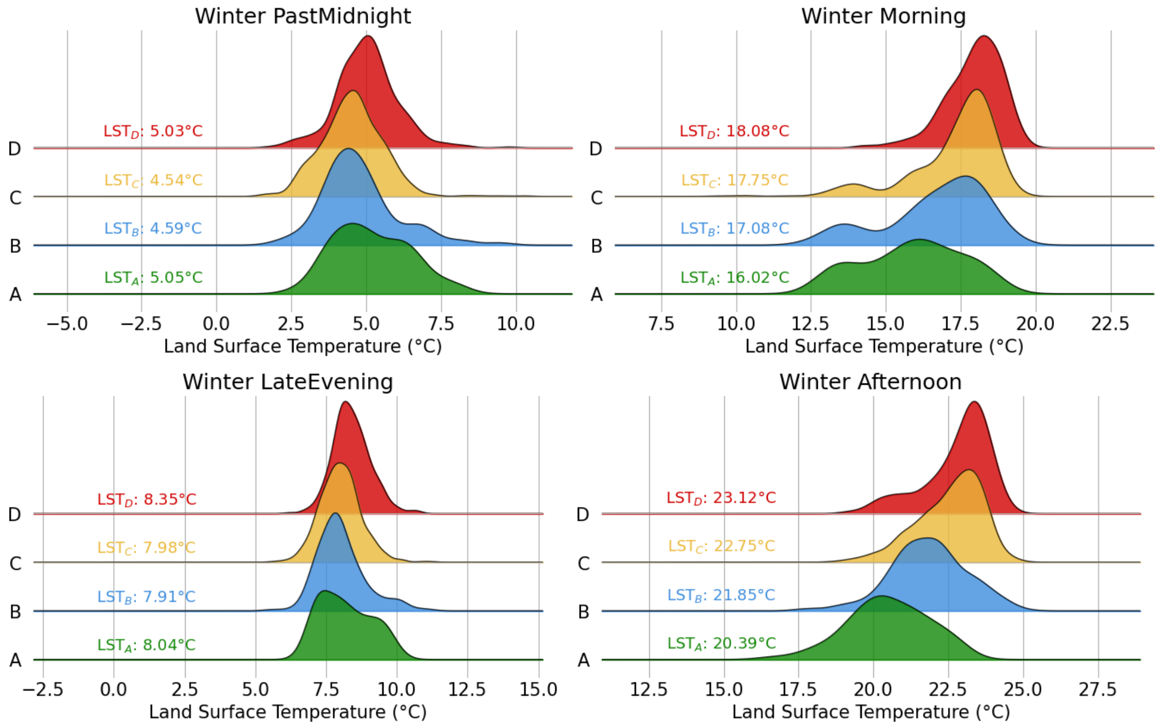

SI Figure 2c: Same as Figure 3 but for the Winter season

Fig 3: Cumulative LST distribution for Summer afternoon

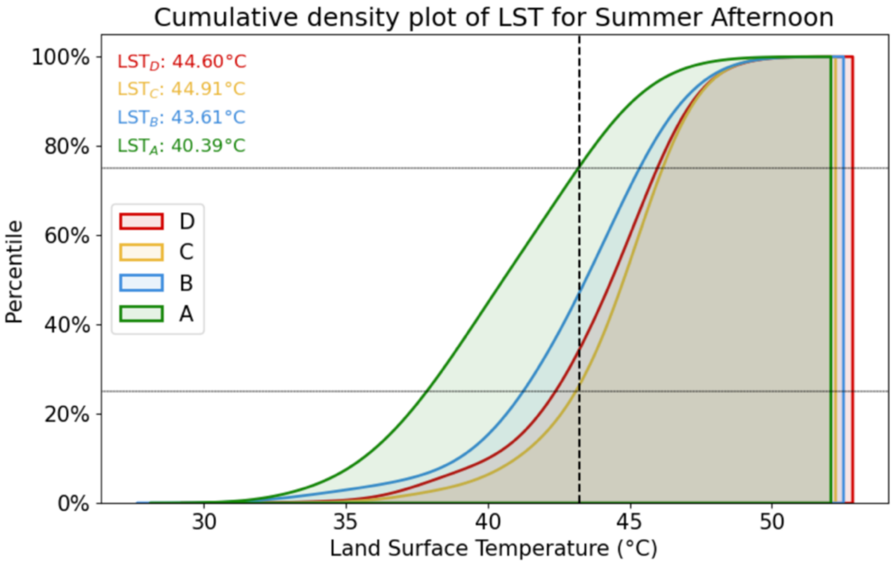

SI Figure 3: Cumulative distribution plot of LST to illustrate the percent of population in each HOLC class (A-D) that experience temperatures above a specific threshold, say 43°C. This threshold corresponds to 75th percentile for class A which means that only 25% of the population experience LSTs above this threshold. On the other hand, the same threshold corresponds to 25th percentile for class C where 75% of the population experience LSTs above this threshold.

Fig 4: Kolmogorov Smirnov test results

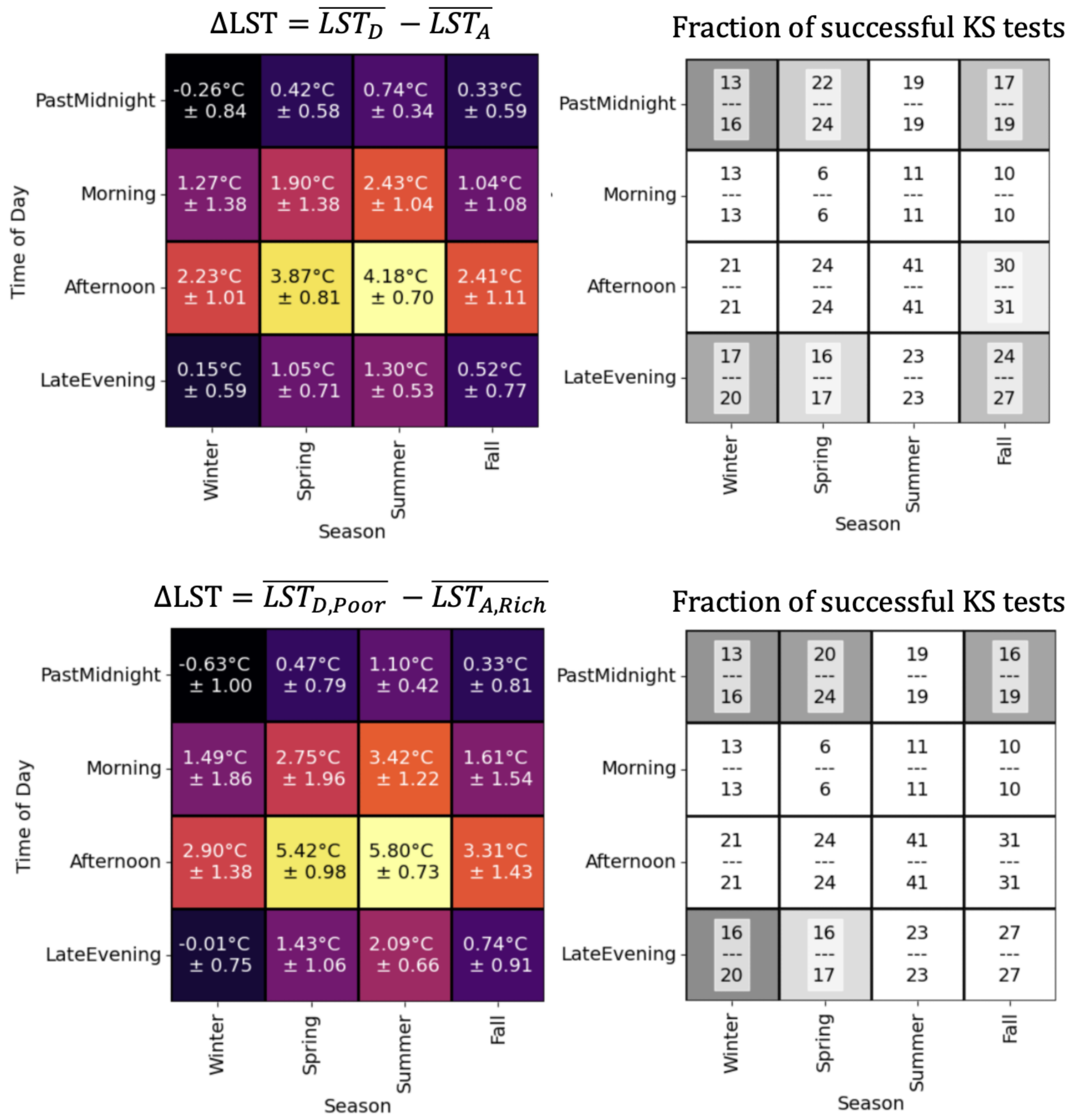

SI Figure 4: (Left) Comparison of the two definitions of thermal disparities (ΔLST) designed to complement Figures 3e and 5f (the brighter colors represent the larger ΔLST and the darker colors represent the smaller ΔLST). We see that across the two definitions, the diurnal and seasonal trends in ΔLST remain the same although the magnitude of change is different. (Right) Fraction of statistically significant ΔLSTs out of the total number of ECOSTRESS scenes for each combination of season and time of day.

Fig 5: Median household income histogram and classification of rich, medium, and poor

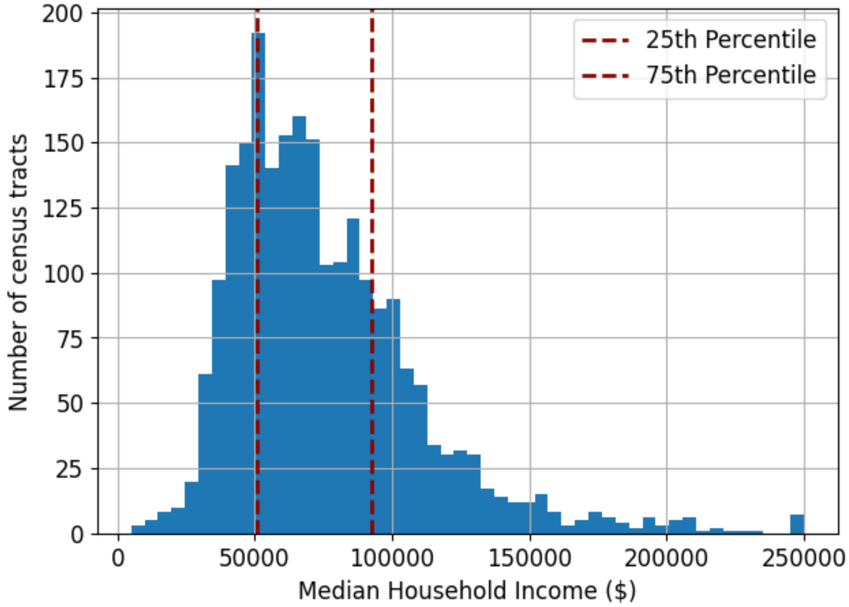

SI Figure 5: Plot of the mean household income as a function of the number of census tracts with the 25th and 75th percentile of income shown as dashed lines (bottom)

Fig 6: Scatter plot between the percentage of households below poverty line and median household income

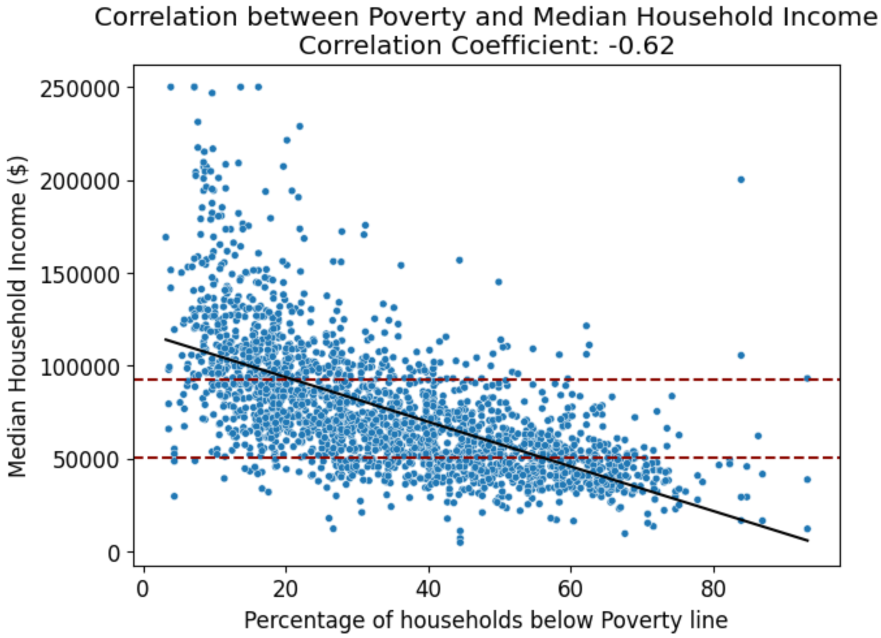

SI Figure 6: Scatter plot between the percentage of households below poverty line and median household income

Fig 7a: LST boxplots by income classes and HOLC grades in Spring

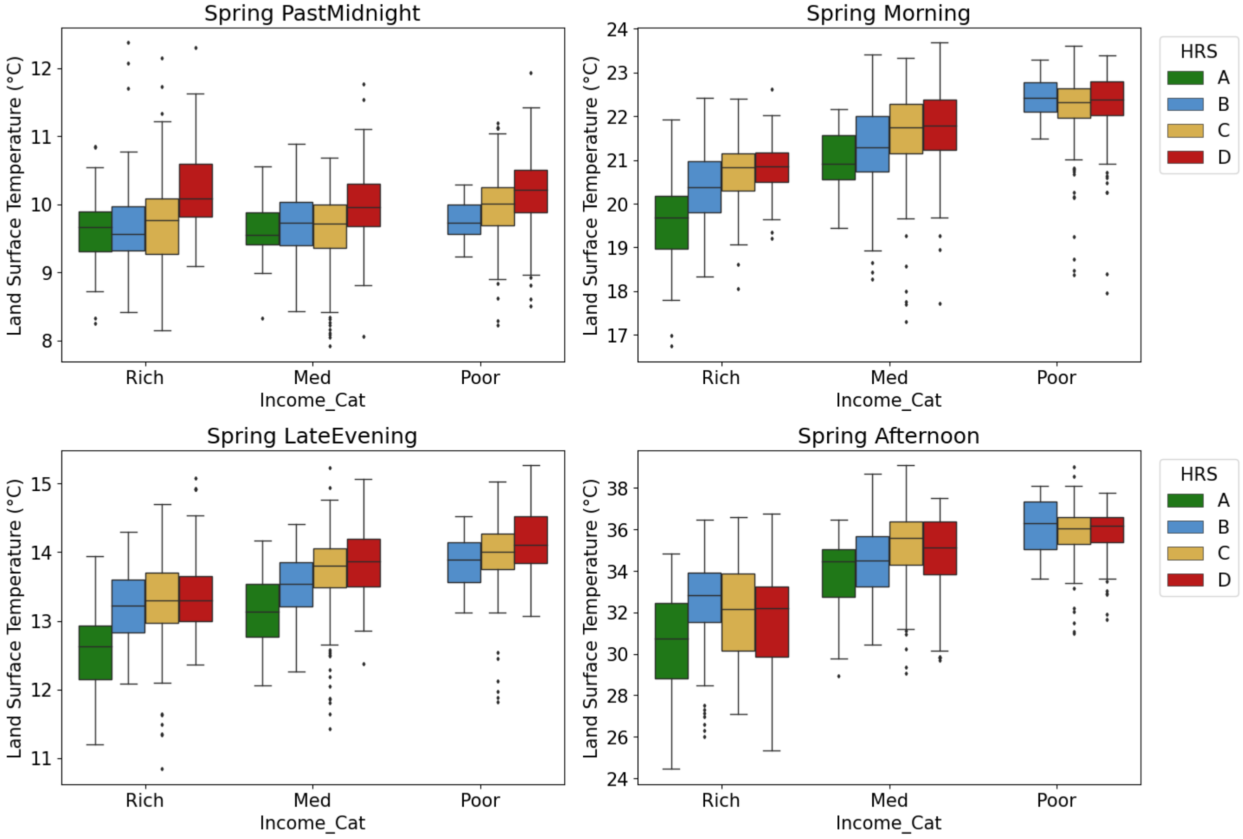

SI Figure 7a: Box-plots of LST organized by the three income categories (rich, medium, poor), and the HOLC classes (A-D) for four time periods (past-midnight, morning, afternoon, late-evening) arranged in clockwise order for Spring season.

Fig 7b: LST boxplots by income classes and HOLC grades in Summer

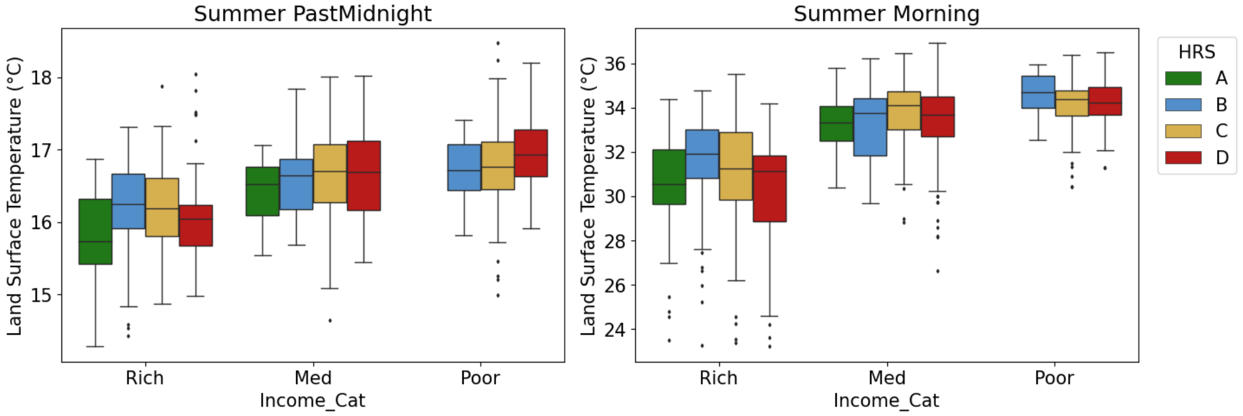

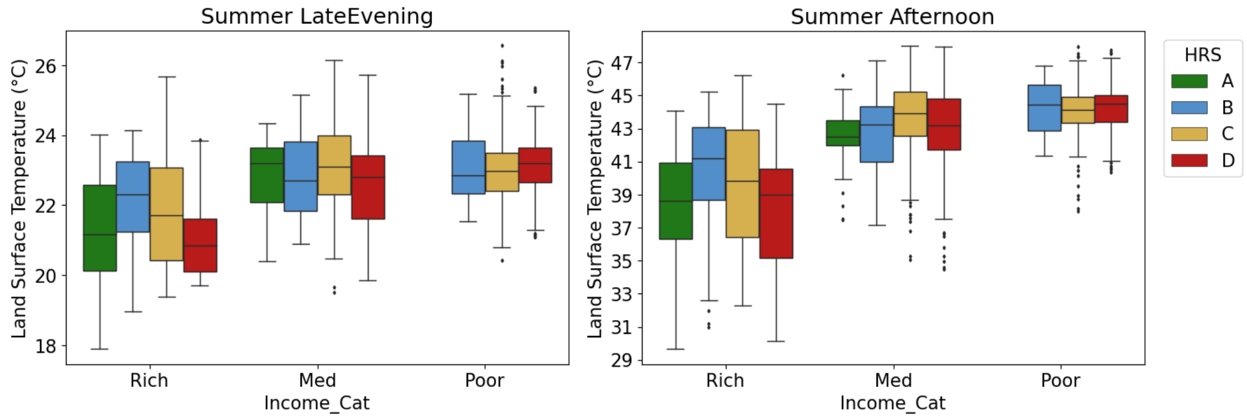

SI Figure 7b: Same as figure 7a for Summer season.

Fig 7c: LST boxplots by income classes and HOLC grades in Fall

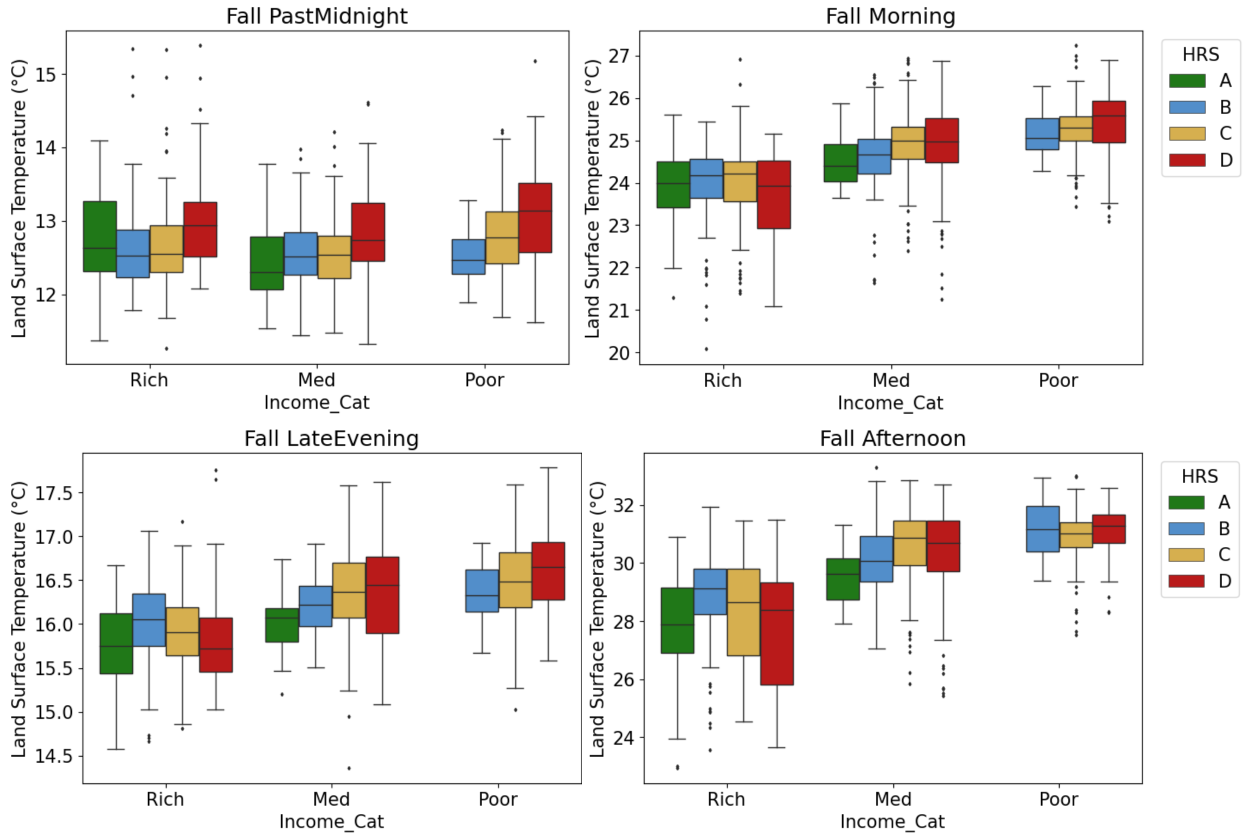

SI Figure 7b: Same as figure 7a for Fall season.

Fig 7d: LST boxplots by income classes and HOLC grades in Winter

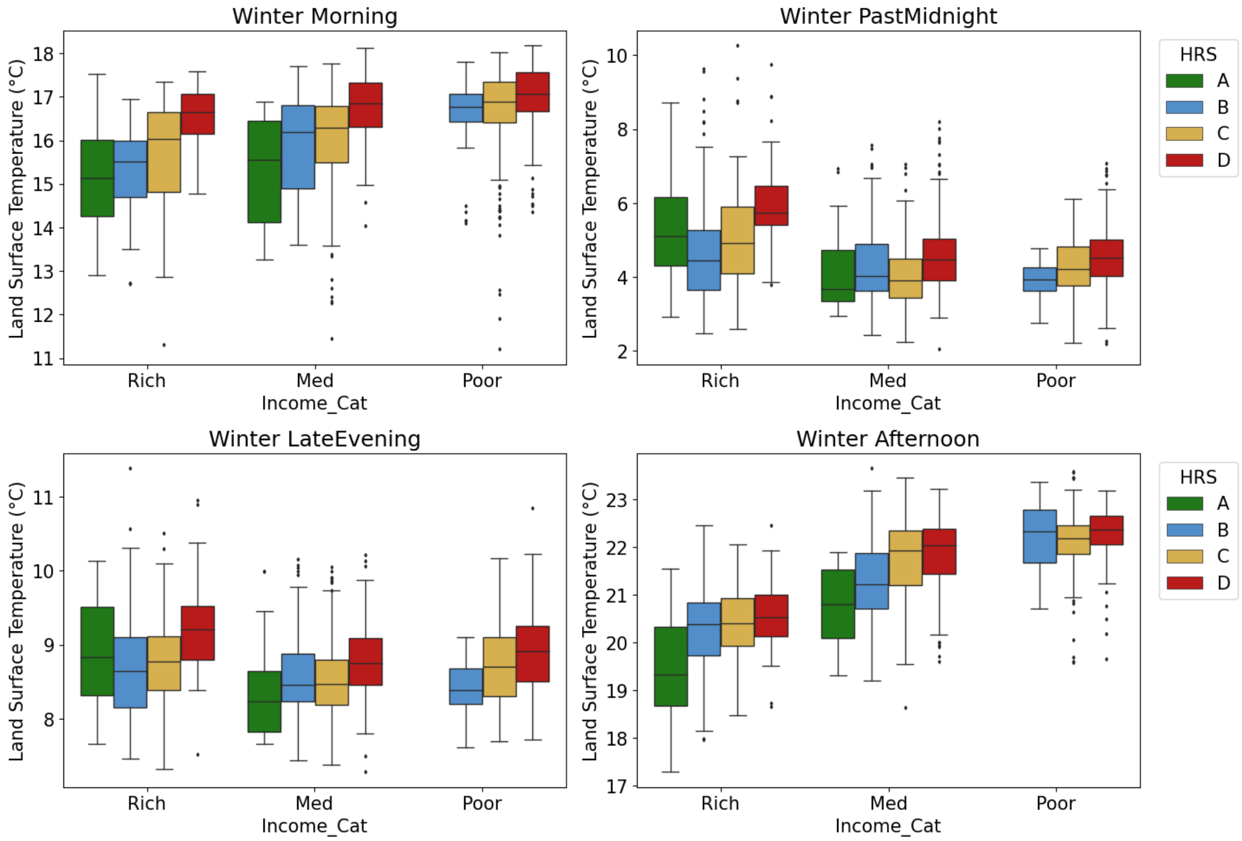

SI Figure 7b: Same as figure 7a for Winter season.

Number of data points used for each combination of Income class and HOLC grade are given below:

	High	Mid	Low
A	57	21	0
B	73	89	32
C	74	356	240
D	45	172	160

Fig 8: Seasonal hysteresis in vegetation indices

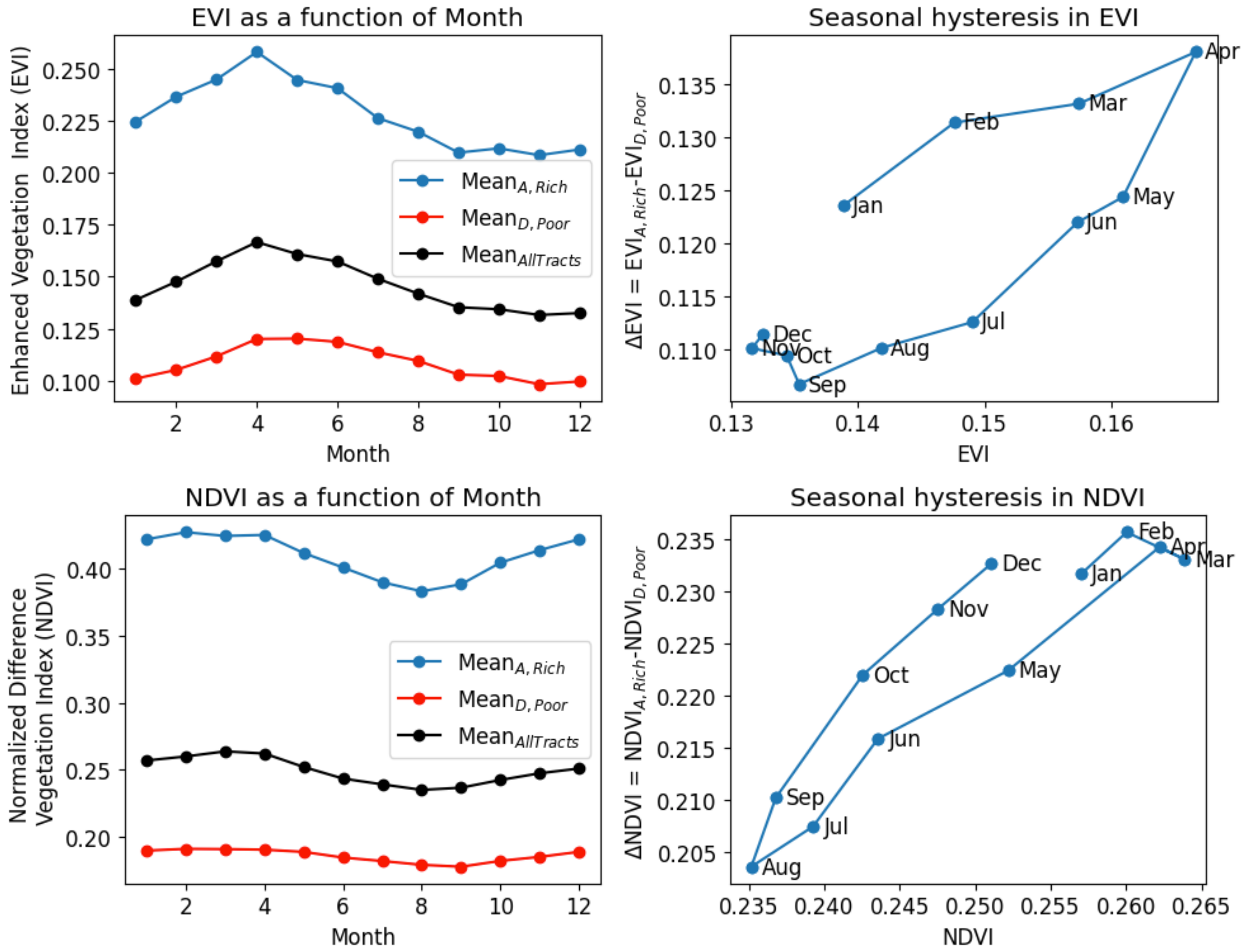

SI Figure 8: (top-left) Monthly average of the Enhanced Vegetative Index (EVI) plotted as a time-series. Each line denotes the spatial mean/median computed for census tracts (A,Rich; D, Poor; and all combined) (top-right) Difference in EVI between the two extreme classes (A, Rich and D, Poor) are shown for each month as a function of mean EVI. The bottom row is same as the top row but for Normalized Difference Vegetation Index (NDVI).

Fig 9: Seasonal hysteresis for each time of day

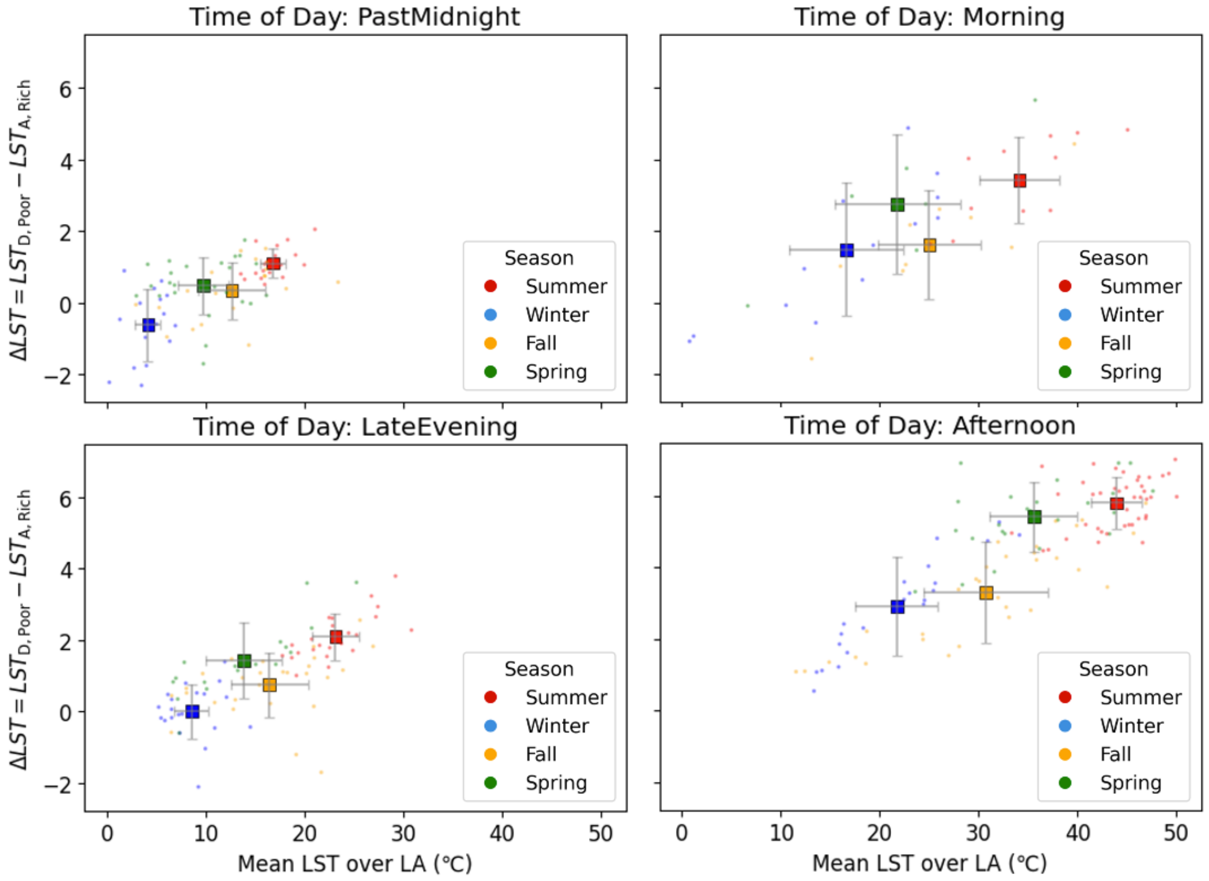

SI Figure 9: Difference in LST between the two extreme classes (D, Poor and A, Rich) plotted as function of temperature (std. dev. is shown as error bars). The colors represent the four seasons, and the four panels represent the four times of day presented clockwise. The number scenes used for each as given below:

(a)

Season	Winter	Spring	Summer	Fall
**Time_of_day**				
**PastMidnight**	18	24	21	21
**Morning**	14	6	12	12
**Afternoon**	22	26	44	33
**LateEvening**	21	19	23	29

Fig 10: Air temperature distributions across the HOLC grades

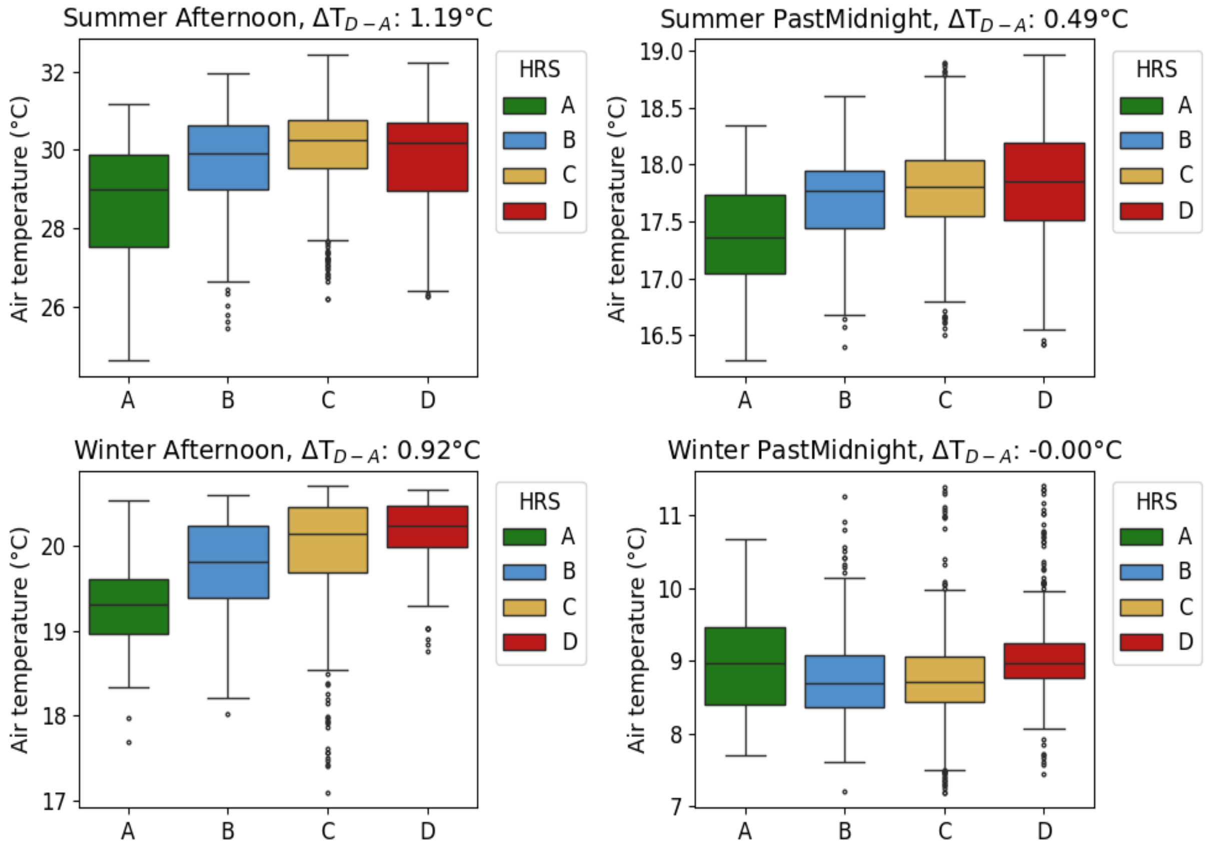

SI Figure 10: Box plots of model-derived 2m air temperatures for each of the four HOLC classes (A-D). The difference between the air temperatures across classes A and D are highlighted in the figure panel titles. The sample size of census tracts (n) in each of the HOLC grades are as follows: $n_A = 78$, $n_B = 195$, $n_C = 670$, and $\$n_D = 377$. Box plots show medians (center lines), interquartile ranges (boxes), 5th-95th percentiles (whiskers), and outliers (points).

Fig 11a: Principal Component Analysis

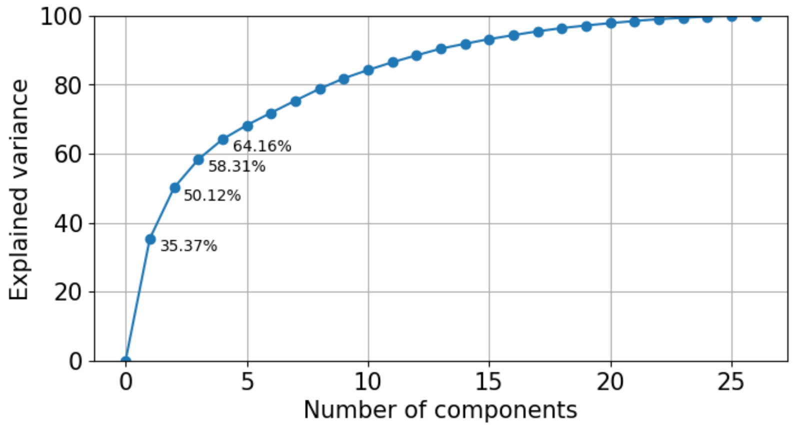

SI Figure 11a: Plot of the cumulative variance explained (in percent) as a function of the number of Principal Components.

Fig 11b: PCA loading of each variable for first 3 PCs.

Variable name	PC1	PC2	PC3	Category	Description	Source
LessThanHS	0.89	-0.18	-0.09	Literacy	% population over age 25 without high school education	ACS
MaxEd_HS	0.61	-0.39	0.11	Literacy	% population over age 25 without education past high school	ACS
‘MedianYear	-0.23	-0.31	-0.27	Living conditions	Average age of build of dwellings	ACS
ForeignBorn	0.47	0.00	-0.28	Isolation	% foreign-born population	ACS
Elderly	-0.54	0.01	0.63	Health and age	% population over age 65	ACS
Young	0.29	-0.27	-0.14	Health and age	% population under age 5	ACS
OutdoorWorkers	0.71	-0.34	-0.10	Occupation	% workers employed in construction, extraction, and maintenace	ACS
Unemployment	0.33	0.26	0.18	Occupation	% unemployed out of working population	ACS
Renting	0.47	0.65	-0.38	Living conditions	% renter-occupied housing units	ACS
ManyOccupants	0.64	-0.59	-0.06	Living conditions	% housing units with more than 4 occupants	ACS
Crowding	0.64	0.20	-0.32	Living conditions	% housing units with more than 1.5 occupants per room	ACS

ExtremePoverty	0.36	0.61	0.13	Economic	% households with yearly income less than $10,000	ACS
LivAlone	-0.28	0.83	0.10	Isolation	% householders living alone	ACS
LivAloneOver65	-0.30	0.40	0.61	Isolation	% householders living alone over age 65	ACS
Solar	-0.11	-0.19	0.16	Economic	% housing units that use solar energy	ACS
NoDriveToWork	0.44	0.66	-0.19	Occupation	% wokers that commute via public transportation, cycling or walking	ACS
LongCommute	0.32	-0.16	0.10	Occupation	% wokers with commute times of over 1 hour	ACS
NoVehicle	0.49	0.71	0.11	Isolation	% housing units that do not own a car	ACS
CalPoverty	0.91	0.21	-0.10	Economic	% population living below two times the federal poverty level	CES
HouseBurden	0.72	0.31	-0.09	Economic	% households severly burdened (>50% of income) by housing costs	CES
EnergyBurden	0.76	-0.16	0.22	Economic	Average household energy cost divided by average household income	CEJST
TravelBarriers	0.77	-0.23	0.03	Isolation	Average relative cost and time spent on transportation	CEJST
Asthma	0.77	0.12	0.23	Health and age	Crude prevalence of asthma among adults	CDC
Diabetes	0.77	-0.08	0.43	Health and age	Crude prevalence of diagnosed diabetes among adults	CDC
Obesity	0.88	-0.05	0.15	Health and age	Crude prevalence of obesity among adults	CDC
Stroke	0.64	0.09	0.66	Health and age	Crude prevalence of stroke among adults	CDC

SI Figure 11b: Principal Component Loadings for the first three principal components indicating the relative contribution of each variable on which the PCA is performed. Here, the color coding is as follows: red indicates a positive loading, blue indicates a negative loading, and the strength of the relationship is shown as the degree of shading.

Fig 12: Population percentage of each of the individual ethnicities

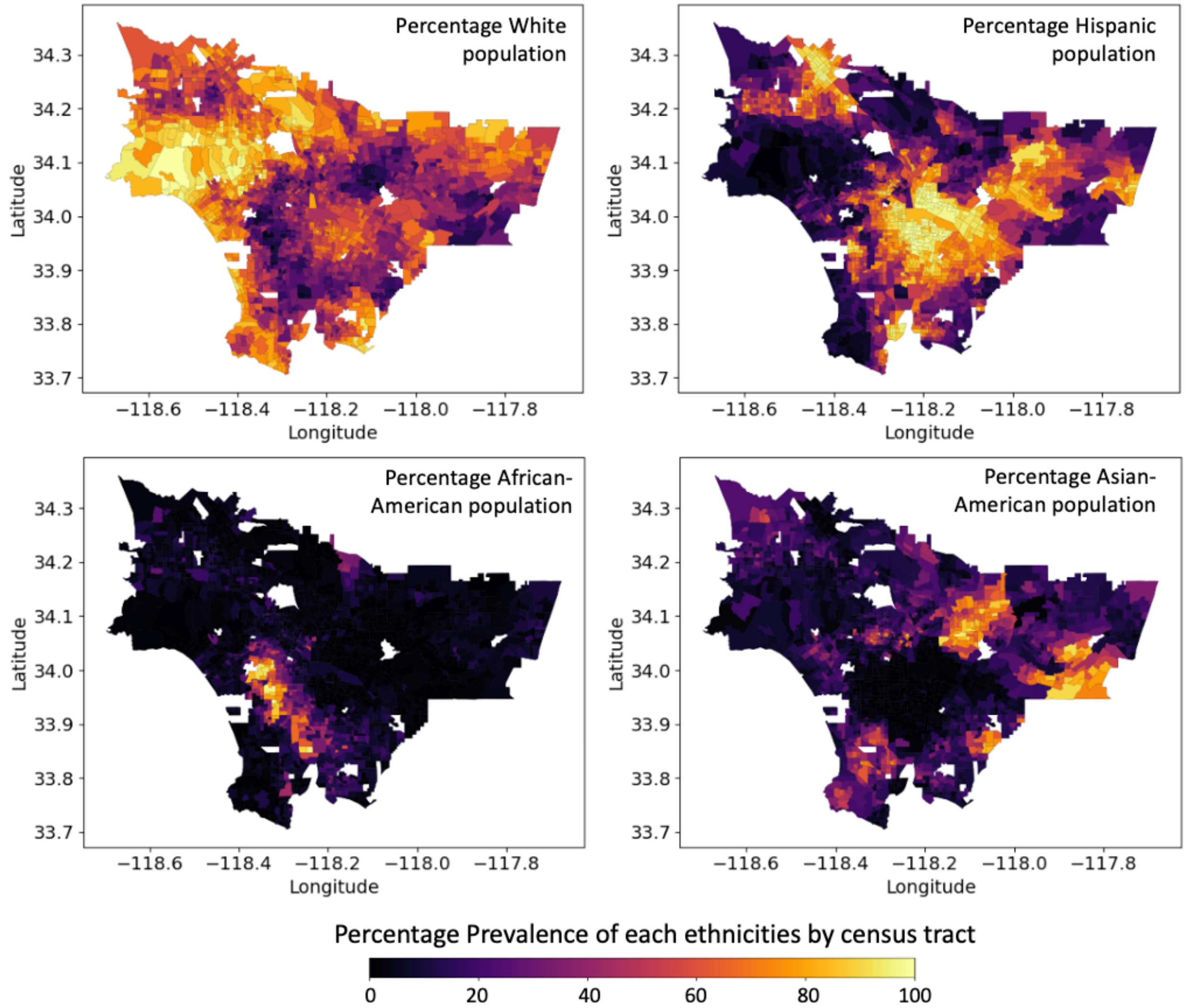

SI Figure 12: Population percentage of the four predominant ethnic groups within each census tract. Census tract boundaries are based on 2020 shapefiles provided by the U.S. Census Bureau.

Fig 13: Vulnerability Score distribution for each race/ethnicity

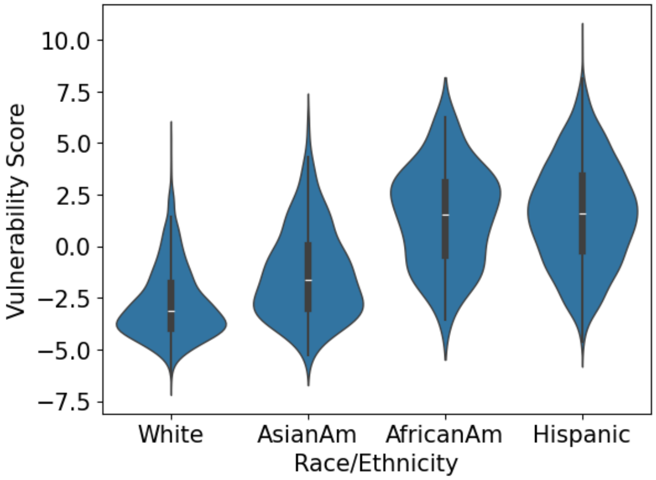

SI Figure 13: Distribution of Vulnerability Score (or PC1) is shown for each of the four major racial/ethnic groups present in LA.

Fig 14: Population percentage sorted by HOLC grade and income

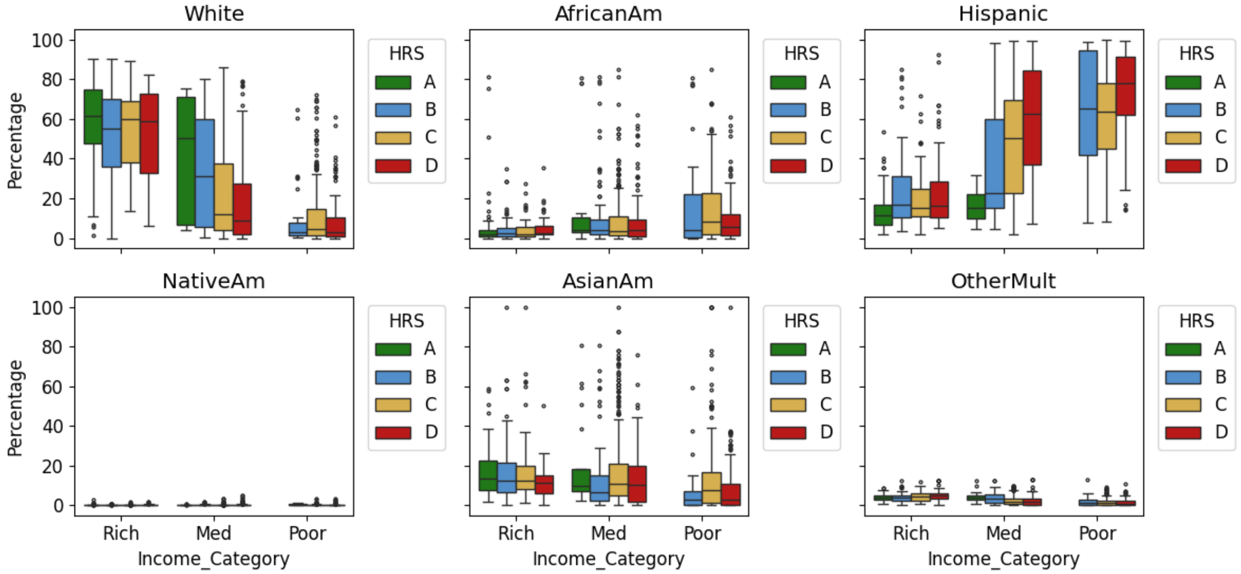

SI Figure 14: Percentage of each ethnic group within the four HOLC grades (A-D, shown in color) and the three income categories (high, medium, low). The sample size of census tracts (n) in each of the HOLC grades are as follows: n_A = 78, n_B = 195, n_C = 670, and \$$n_D$ = 377. Box plots show medians (center lines), interquartile ranges (boxes), 5th-95th percentiles (whiskers), and outliers (points).

Fig 15: Analysis of AC usage data from multiple sources

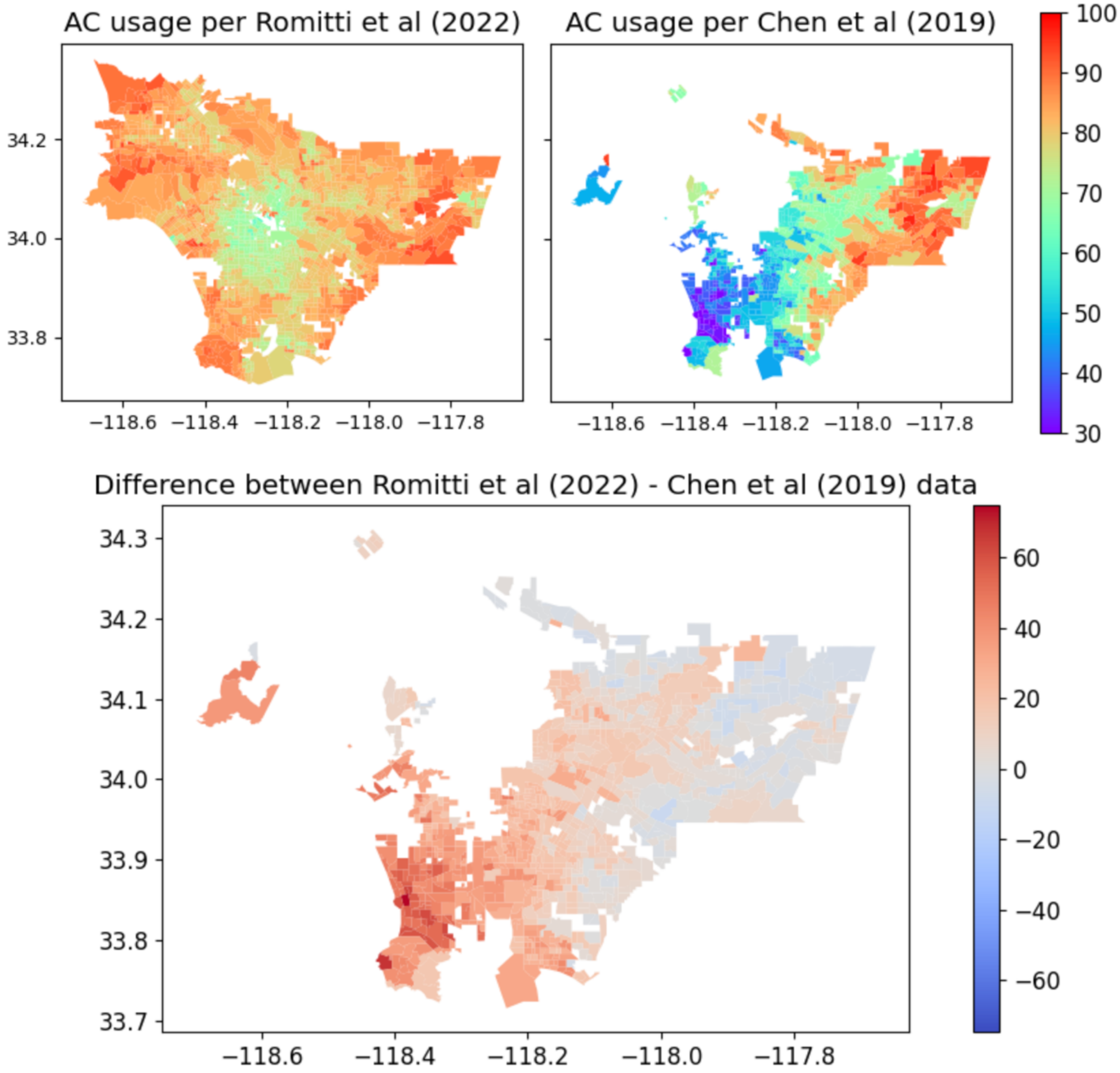

SI Figure 15: Here we illustrate a comparison of AC usage data sourced from two distinct references. The disparity plot, calculated as $AC_{Romitti} - AC_{Chen}$, reveals that the Romitti et al. (2022) method tends to overestimate AC usage, particularly near the coast in LA. This discrepancy arises because Romitti et al. (2022) relies on a model that extrapolates AC usage from diverse demographic factors, climate conditions, and geographic variances. Conversely, Chen et al. (2019) utilizes actual electricity data to ascertain real AC usage over the year, revealing that actual usage along the coastal regions is lower than anticipated due to the ameliorating effect of the coastal breeze. Nonetheless, given that Chen et al. (2019) data does not cover the entire study area, a combined AC estimate from both datasets is utilized. Census tract boundaries are based on 2020 shapefiles provided by the U.S. Census Bureau.
